# Association of breast milk microbiota and metabolites with neonatal jaundice

**DOI:** 10.3389/fped.2024.1500069

**Published:** 2025-01-06

**Authors:** TianYu Liu, Yanhan Yuan, Jinying Wei, Jiayi Chen, Feng Zhang, Juanjuan Chen, Jinping Zhang

**Affiliations:** ^1^ College of Food Science and Technology, Shanghai Ocean University, Shanghai, China; ^2^ Department of Pediatrics, Shanghai Sixth People’s Hospital Affiliated to Shanghai Jiao Tong University School of Medicine, Shanghai, China

**Keywords:** breast milk, breastfeeding, neonatal jaundice, microbiota, metabolite

## Abstract

**Background:**

Breast milk is the primary source of nutrition during early life, and existing research indicates that the development of jaundice in breastfed newborns may be linked to specific nutrients or bioactive substances present in breast milk. However, the association between the microbiota and small-molecule metabolites in breast milk and the development of neonatal jaundice remains unproven. This study aimed to investigate the development of jaundice in breastfed neonates in relation to breast milk microbiota and metabolites.

**Methods:**

Based on the conditions of exclusive breastfeeding, we selected healthy newborns without significant jaundice and their mothers on day 4 (96–120 h after birth) as the healthy control group, and jaundiced newborns and their mothers as the jaundice group. Breast milk samples were collected from mothers in both groups on postnatal day 4 and analyzed for microbiota and small-molecule metabolites using 16S rRNA gene sequencing and an liquid chromatography-tandem mass spectrometry techniques.

**Results:**

A total of 104 mother-child pairs were included in the study, of which 51 pairs were in the healthy control group and the other 53 pairs were in the jaundice group. Our results demonstrated that there was no significant difference between the species composition and diversity of the breast milk flora in the healthy control and jaundice groups. At the genus level, the abundance of *Lactobacillus*, *Ackermannia*, and *Bifidobacterium* was significantly higher in the breast milk of the healthy control group than in the jaundice group. Metabolomics analysis revealed a total of 27 significantly different metabolites between the two groups. Notably, breast milk from the healthy control group had elevated levels of 24 metabolites, predominantly lipids family, including sphingolipids, phospholipids, and fatty acid derivatives.

**Conclusion:**

This study suggests that there is a link between the development of neonatal jaundice and breast milk microbiota and metabolites. Breast milk from mothers of healthy newborns contains higher levels of beneficial bacteria and lipid family compared to mothers of newborns with jaundice. This study offers new insights into the relationship between breastfeeding and neonatal jaundice.

## 1 Introduction

Neonatal jaundice is one of the most common clinical issues in the neonatal period, affecting approximately 60% of term infants and 80% of preterm infants (1). A recent clinical meta-analysis reported that the prevalence of severe neonatal jaundice varies between 0.73% and 3.34% across different World Health Organization (WHO) regions, indicating its significance as a global public health concern (2). Elevated concentrations of unconjugated bilirubin can cross the neonatal blood-brain barrier, bind to brain tissue and induce neurotoxicity, leading to bilirubin encephalopathy or brain damage, which may potentially cause long-term neurological sequelae and adversely impact the neonate's overall health (3). Therefore, developing effective strategies for the prevention and treatment of neonatal jaundice has become a public health priority. In our clinical practice, we have observed variability in jaundice development among breastfed newborns, raising the question of whether it is related to the microbiota and small-molecule metabolites present in breast milk, which is the central focus of our study.

Breast milk is the optimal natural food for newborns during the early stages of life, fully meeting their nutritional requirements for growth and development (4). The WHO recommends exclusive breastfeeding for the first six months of life, with continued breastfeeding alongside complementary foods until at least two years of age to support optimal growth and development (5). As an important link between mother and newborn, breast milk plays a significant role in preventing early disease and supporting the long-term growth and development of the newborn. In addition to macronutrients such as proteins, fats, and carbohydrates, breast milk contains various bioactive substances, including oligosaccharides, microbial components, and small molecules (6). These substances are transferred to the newborn through breastfeeding, where they interact with commensal gut bacteria and immune cells, collectively promoting healthy gut development during the early neonatal period (7). As our understanding of breast milk properties has advanced, its numerous benefits have become increasingly recognized. Breast milk not only provides essential nutrition for newborns but also supports the development and maturation of their digestive, immune, and nervous systems. Furthermore, it has the potential to reduce the risk of infections, allergies, and asthma in newborns (8). Neonatal jaundice is a common condition with a higher incidence compared to other neonatal issues. Its aetiology may involve multiple factors, including physiological, autoimmune (Rh sensitisation), genetic and environmental factors (9).Traditionally, breastfeeding has been associated with neonatal jaundice; however, no definitive composition of breast milk accounts for all cases of jaundice (10). It has been proposed that a reduction in short-chain fatty acids (SCFAs) due to dysbiosis of the intestinal flora may contribute to breastfeeding jaundice. Additionally, discontinuing breastfeeding to treat jaundice might increase the risk of adverse health outcomes during neonatal growth (11). Recent research indicates that exclusive breastfeeding during the first six months may help mitigate the adverse neurological effects associated with severe neonatal jaundice (12). These studies suggest that the benefits of breast milk and breastfeeding outweigh the risks associated with jaundice. A systematic review has identified associations between various nutrients (e.g., fats, proteins, lactose, and minerals) and biologically active constituents (e.g., enzymes, bile salts, cytokines, epidermal growth factors, and steroids) in breast milk and the development of neonatal jaundice (13). However, the potential link between the microbiota and small-molecule metabolites in breast milk and the development of neonatal jaundice remains to be demonstrated.

Neonatal jaundice occurs as a result of elevated serum bilirubin levels due to excessive production of bilirubin in the body and abnormal bilirubin metabolism and excretion. The gut is a crucial pathway for bilirubin metabolism, and increased enterohepatic circulation of bilirubin is a key factor in the development of neonatal jaundice. Studies have shown that the intestinal microbiota is involved in bilirubin metabolism and that its composition is closely related to serum bilirubin levels (14). Dysbiosis of the intestinal microbiota interferes with the normal conversion process of bilirubin in the body, leading to increased enterohepatic circulation of bilirubin, which in turn triggers jaundice (15–17). Infants with jaundice usually show characteristic changes in the composition of the gut microbiota compared to healthy infants, which may be manifested by an increase in the abundance of *Klebsiella* or a decrease in the abundance of *Bifidobacterium* (18–20). Another case-control study showed an increased abundance of Bacteroidota in the intestinal flora of children with pathological jaundice compared to healthy newborns (21). Additionally, intestinal inflammation can induce intestinal microbial dysbiosis, and this dysbiosis can exacerbate the response to severe intestinal inflammation. The interaction between these factors can lead to decreased intestinal motility, which may increase enterohepatic recycling of bilirubin and, ultimately, cause neonatal jaundice (22, 23). Therefore, maintaining a healthy intestinal environment in newborns is essential for preventing jaundice.

In breastfed newborns, certain bioactive components of breast milk have anti-inflammatory effects and are involved in regulating the establishment of intestinal flora, which may help protect against neonatal jaundice. Breast milk plays a critical role in establishing healthy gut flora in newborns. By one month of age, approximately 25%–30% of the neonatal gut microbiota originates from breast milk (24). Beneficial bacteria in breast milk, notably *Lactobacillus* and *Bifidobacterium*, are transmitted to newborns through breastfeeding and possess antibacterial, anti-inflammatory, anti-infectious, and immunomodulatory properties (25). Additionally, some small-molecule metabolites in breast milk can be metabolized in the intestine into products that act as signaling substances and intermediate metabolites, playing roles in metabolic processes as well as in immunomodulation and attenuation of inflammatory responses (7). For instance, tryptophan, a metabolite present in breast milk, has been shown to influence host-microbiota interactions and modulate the immune response of CD4+ cells and monocytes in the gut in a dose-dependent manner, thereby promoting early immune system development (26). Moreover, omega-3 polyunsaturated fatty acids in breast milk not only reduce the production of inflammatory cytokines but also increase the abundance of anti-inflammatory microorganisms, such as *Lactobacillus* and *Bifidobacterium*, in the neonatal gut, which helps alleviate intestinal inflammation (27, 28). These studies have shown that beneficial microorganisms and small-molecule metabolites in breast milk may contribute to some degree to the healthy development of the neonatal gut.

However, the association between these substances and the development of neonatal jaundice requires further investigation. This study aimed to explore the relationship between microbiota and small-molecule metabolites in breast milk and neonatal jaundice by collecting breast milk samples from breastfeeding mothers and analyzing them using 16S rRNA gene sequencing and LC-MS techniques.

## 2 Methods and analysis

### 2.1 Study participants

**Figure 1:**
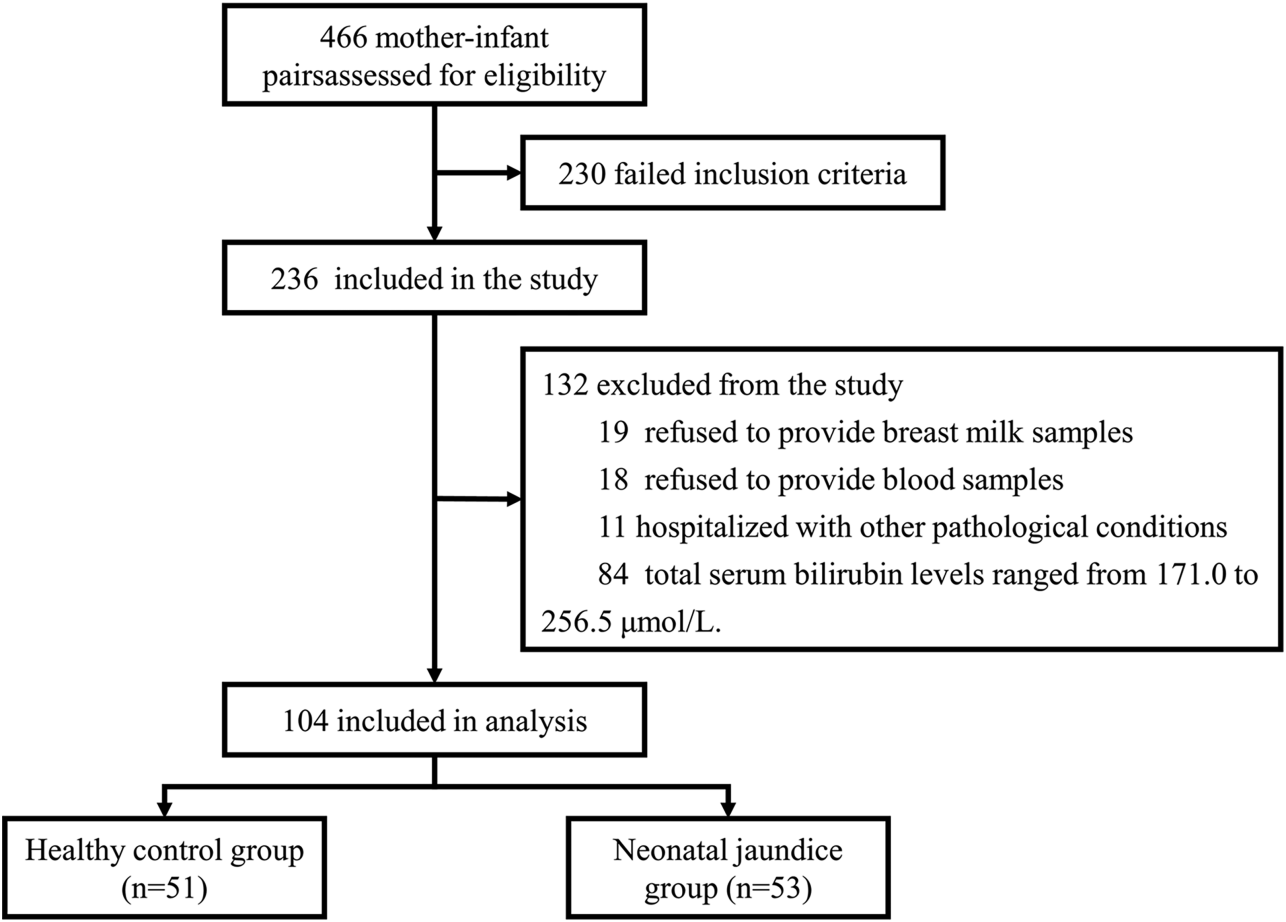
Flow diagram.

Subjects were recruited from March 2023 to March 2024 from the Department of Obstetrics and Gynaecology and the Department of Paediatrics at Shanghai Sixth People's Hospital. The whole process is represented as a flow diagram (Figure 1). All infants were exclusively breastfed from birth. We measured the total serum bilirubin (TBIL) levels of the newborns on day 4 of life (96–120 h after birth). The grouping criteria in this study were based on the neonatal hourly bilirubin column diagram developed by Bhutani et al. (29) in the United States and the grouping criteria of the study by Akagawa et al. (16). Newborns with serum TBIL concentrations exceeding 256.5 μmol/L were classified into the neonatal jaundice group (NJ), while those with concentrations below 171.0 μmol/L were classified into the healthy control group (HC). The study adhered to the principles outlined in the Declaration of Helsinki and was approved by the Ethics Committee of Shanghai Sixth People's Hospital [Ethical Approval Number: 2023-122-(1)]. Additionally, the study was registered with the China Clinical Trial Center (Registration Number: ChiCTR2300076544). Informed consent was obtained from all participants.

### 2.2 Inclusion and exclusion criteria

The inclusion criteria for this study were as follows: (1) newborns were required to have a gestational age between 37 and 42 weeks and a birth weight ranging from 2,500 g to 4,000 g; (2) needed to be exclusively breastfed; (3) the mothers should be between 20 and 35 years of age, with a pre-pregnancy body mass index (BMI) between 18.5 kg/m^2^ and 24.9 kg/m^2^; (4) Mothers with low-risk pregnancies.

The exclusion criteria were as follows: (1) newborns with rapidly increasing bilirubin levels that necessitated phototherapy within the first 3 days after birth were excluded; (2) mothers or newborns who had used antibiotics, probiotics, or other micro-ecological agents during breastfeeding; (3) newborns with bilirubin levels meeting the criteria for blood exchange; (4) newborns suffering from other pathological conditions, including congenital immunodeficiencies, congenital biliary or other organ malformations, abnormal liver function, or enteric diseases; (5) Mothers and their newborns who give birth using vacuum extraction or forceps.

### 2.3 Collection of clinical information

Basic demographic and clinical information, including maternal age, pre-pregnancy BMI, mode of delivery, gestational age of the newborn, sex, and birth weight, was collected through online retrieval of medical records from the hospital system and offline face-to-face communication.

### 2.4 Blood collection and testing

On the fourth day of life, 1 ml of peripheral venous blood was collected from each newborn. Allow to stand at room temperature for 30 min, then centrifuge at 3,000 rpm for 15 min at 4°C. The supernatant was transferred to sterile, enzyme-free centrifuge tubes, and the total serum bilirubin level was measured using a fully automated chemical analyzer (Beckman Coulter, AU588, USA).

### 2.5 Breast milk sample collection

Breast milk samples were collected from lactating mothers on the fourth postpartum day, and all samples were collected between 9:00 and 11:00 am. Collect foremilk samples from unilateral breasts that have not fed for 2 h. To minimise contamination from external bacteria during breastfeeding, the investigator, wearing sterile gloves, first cleaned the subject's nipples and surrounding skin thoroughly using sterile saline-soaked gauze (30). Subsequently, 5–10 ml of milk was hand-expressed from one breast into a 15 ml sterile tube. The milk was gently mixed and immediately placed in a dry ice box for transport. The samples were then frozen at −80°C for preservation and transported in a dry ice transfer box to Shenzhen Micro-max Technology Group Limited for DNA extraction and sequencing.

### 2.6 Breast milk microbiota analysis

DNA was extracted from breast milk samples using the cetyltrimethylammonium bromide (CTAB) method. The purity and concentration of the DNA was then tested using 1% agarose gel electrophoresis by taking an appropriate amount of sample in a centrifuge tube and diluting the sample to 1 ng/µl using sterile water. The V3-V4 variable region of the 16S rRNA gene was ampli-fied using the primers 338F (5′-ACTCCTACGGGAGGCAGCAG-3′) and 806R (5′-GGACTACHVGGGTWTCTAAT-3′) via polymerase chain reaction (PCR). All PCR reactions were performed using 15 µl Phusion® High-Fidelity PCR Master Mix (New England Biolabs), 2 µm forward and reverse primers and approximately 10 ng template DNA. Thermal cycling consisted of an initial denaturation at 98°C for 1 min, followed by 30 cycles of denaturation at 98°C for 10 s, annealing at 50°C for 30 s, extension at 72°C for 30 s, and a final extension at 72°C for 5 min. The PCR products were mixed in aliquots according to the concentration of the PCR product, and after thorough mixing, the PCR products were purified by agarose gel electrophoresis using 1XTAE at 2% concentration, and the target bands were recovered using the Universal DNA Purification and Recovery Kit (TianGen, China).

The library was constructed using the NEB Next® Ultra DNA Library Prep Kit (Illumina, USA), and the constructed library was detected and quantified by Q-PCR using the Agilent 5,400 (Agilent Technologies Co Ltd., USA); after library qualification, the library was sequenced using the Illumina sequencing platform. The analyses were performed following the tutorial “Atacama soil microbiome tutorial” in the Qiime2 documentation (https://docs.qiime2.org/2019.1/). The raw sequence FASTQ files were imported into a file format suitable for subsequent QIIME2 processing, using the qiime tools import plugin. The QIIME2 dada2 plugin was then applied for quality control, trimming, denoising, splicing and chimera removal to obtain the final sequence table (31). Next, the QIIME2 feature classifier plugin was applied to compare the representative sequences of ASVs with the Silva 132 database, and a taxonomic information table for the species was obtained (32). The QIIME2 feature table plug-in was then used to remove all contaminating mitochondrial and chloroplast sequences.

### 2.7 Analysis of breast milk metabolites

A 100 μl aliquot of breast milk was placed into a centrifuge tube, and 400 μl of 80% methanol solution was added. The sample was vortexed, incubated in an ice bath for 5 min, and then centrifuged at 15,000 g for 20 min at 4°C. The supernatant was further diluted with LC-MS grade water to achieve a methanol content of 53%. Following a second centrifugation at 15,000 g for 20 min at 4°C, the supernatant was collected and fed into an LC-MS system for analysis.

Ultra-high performance liquid chromatography-mass spectrometry (UHPLC-MS) analyses were performed using a Vanquish ultra-high performance liquid chromatography system (ThermoFisher, Germany) and an Orbitrap Q Exactive™ HF mass spectrometer (Thermo Fisher, Germany). Samples were injected on a HypersilGold column (100 × 2.1 mm, 1.9 μm) with a linear gradient at a flow rate of 0.2 ml/min for 12 min. The eluents for the positive and negative polarity modes were Eluent A (0.1% FA in water) and Eluent B (methanol) respectively: 2% B, 1.5 min; 2%–85% B, 3 min; 85%–100% B, 10 min; 100%–2% B, 10.1 min; and 2% B, 12 min. The Q Exactive™ HF mass spectrometer was operated in positive/negative polarity mode with a beam voltage of 3.5 kV, a capillary temperature of 320°C, a sheath gas flow rate of 35 psi, an auxiliary gas flow rate of 10 L/min, an S lens RF level of 60 and an auxiliary gas heater temperature of 350°C. Raw data files generated by UPLC-MS were processed using Compound Discoverer 3.3 (CD3.3, ThermoFisher) for peak alignment, peak extraction and quantification of each metabolite. Metabolites were then processed and pathways resolved using the KEGG database.

### 2.8 Statistical analysis

As this was exploratory study, we did not calculate the minimum sample size required to achieve a specific statistical power (33–35). Demographic variables were analyzed using independent samples *t*-tests or chi-squared tests. For microbiomic data, differences in alpha diversity indices were compared using the Wilcoxon rank sum test. Differences in microbial community structure between samples were assessed using Jaccard distance and visualized with principal coordinate analysis (PCoA). Beta diversity differences between groups were calculated using permutation multivariate analysis of variance (PERMANOVA). Bacterial community composition was analyzed at the taxonomic level using the Gplots package in R. Potential microbial markers were identified using the linear discriminant analysis effect size (LEfSe) tool. Metabolomics data were analyzed with the R package MetaboAnalystR. Partial Least Squares Discriminant Analysis (PLS-DA) and *t*-tests in univariate analysis were employed to distinguish metabolite differences between groups. Differential metabolites were identified based on fold change (FC) ≥ 2 or FC ≤ 0.5, VIP > 1, and *P*-value <0.05. Volcano plots were generated using the ggplot2 pack-age in R Statistical Software.

## 3 Results

### 3.1 Clinical information of subjects

A total of 104 mother-infant pairs participated in the study, including 51 pairs in the healthy control group and 53 pairs in the jaundice group. Table 1 provides an overview of the general clinical data for these participants. We compared the basic clinical information, including maternal age, prenatal BMI, mode of delivery, neonatal sex, gestational age, and birth weight, between the two groups using one-way ANOVA. No significant differences were observed for these indicators. However, there was a significant difference in serum total bilirubin levels on day 4 between the two groups (*P* < 0.05).

**Table 1:** Characteristics of maternal and infant participants.

Characteristic	HC (*n* = 51)	NJ (*n* = 53)	*P*-value
Mothers’ age (Year)	29.86 ± 3.70	31.06 ± 4.31	0.134
Pre-pregnancy BMI (kg/m^2^)	22.13 ± 1.25	22.46 ± 1.52	0.222
Delivery mode (Natural: C-section)	29:22	29:24	0.826
Gender (Males: Females)	30:21	28:25	0.538
Gestational age (day)	274.47 ± 9.15	272.79 ± 7.49	0.308
Birth weight (g)	3,328 ± 365	3,355 ± 437	0.738
TBIL (μmol/L)	136.58 ± 12.21	276.04 ± 9.90	<0.01

### 3.2 Analysis of breast milk microbiota

#### 3.2.1 Analysis of the diversity of the breast milk microbiota

**Figure 2:**
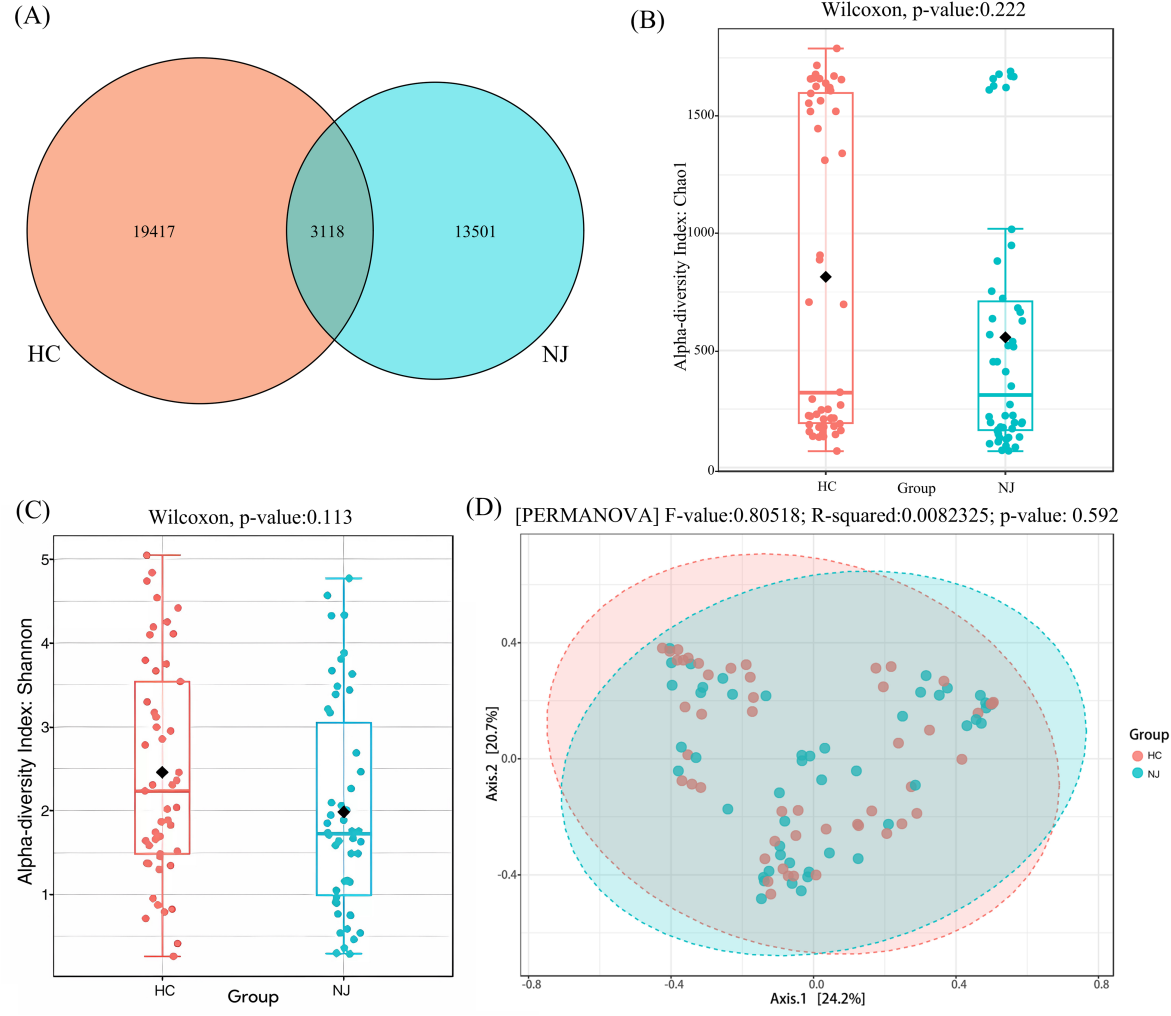
Operational classification units (OTUs), alpha and beta diversity in healthy control (HC) and neonatal jaundice (NJ) groups. **(A)** Venn diagram of operational taxonomic units (OTUs) in the HC and NJ groups. Numbers of unique and shared OTUs in the breastmilk microflora of the HC and NJ groups. **(B)** Chao1 index for HC and NJ groups. **(C)** Shannon diversity index for HC and NJ groups. **(D)** Principal coordinate analysis (PCoA) for β-diversity at the genus level. Each point represents a sample. Orange points represent the HC group, while blue points represent the NJ group.

The results revealed differences in the number of operational taxonomic units (OTUs) between the two groups through 16S rRNA gene sequencing of breast milk samples. A total of 36,036 OTUs were identified across the samples from both the HC and jaundice (NJ) groups. Of these, 3,118 OTUs were shared between the groups, while 19,417 OTUs were specific to the HC group, representing 53.88% of the total OTUs, and 13,501 OTUs were specific to the NJ group, accounting for 37.47% of the total OTUs (Figure 2A). Alpha diversity, assessed using the Chao1 and Shannon indices, was higher in the HC group compared to the NJ group, indicating greater species richness and diversity in the HC group. However, these differences were not statistically significant (Figures 2B,C). Principal Coordinate Analysis (PCoA) of beta diversity revealed that the composition of the breast milk microbiota was structurally similar between the two groups, with no significant differences observed (Figure 2D).

#### 3.2.2 Analysis of the structural composition of breast milk microbiota

**Figure 3:**
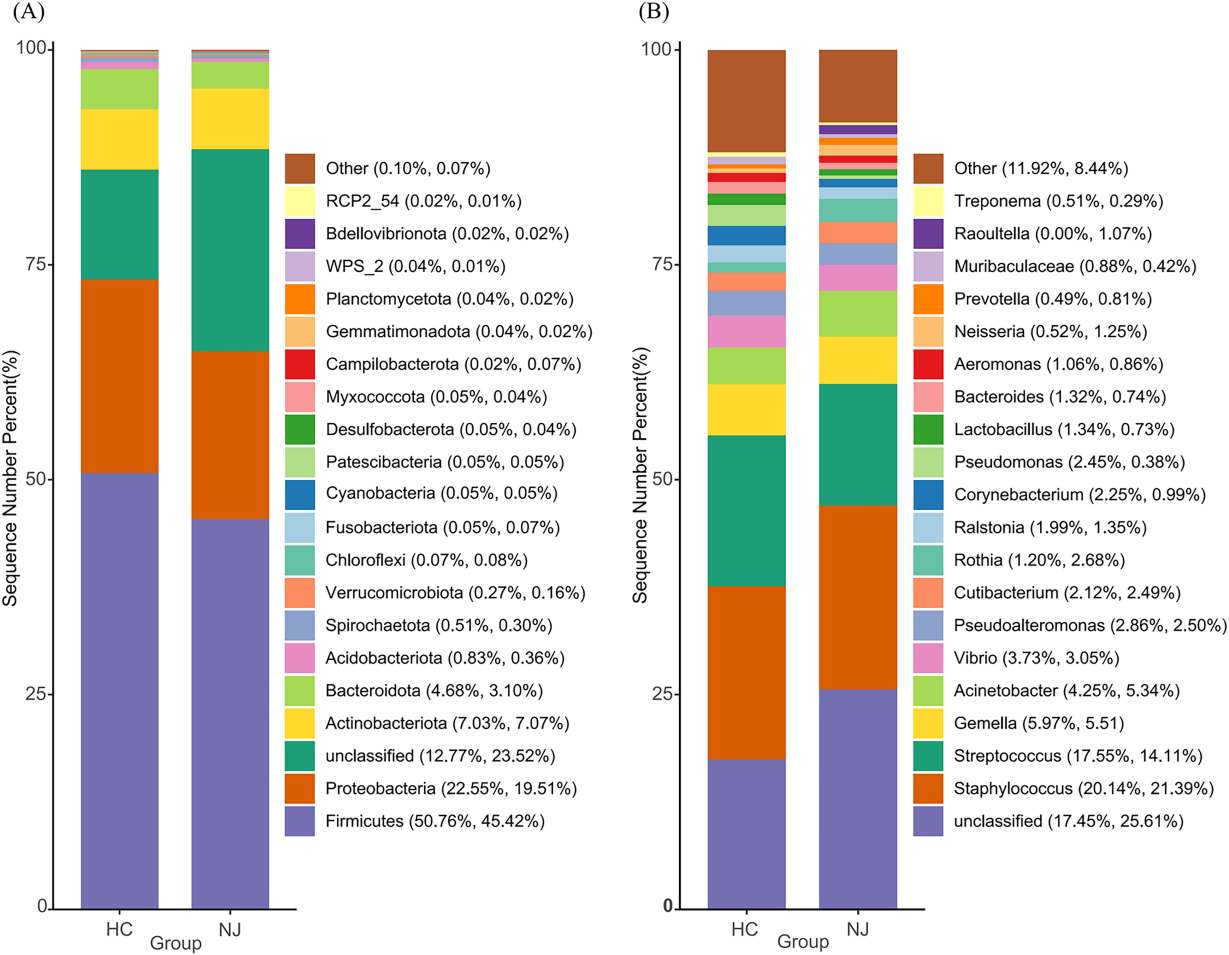
Histograms of relative abundance of the top 20 species at the phylum **(A)** and genus **(B)** levels of breast milk microbiota in the healthy control (HC) and neonatal jaundice (NJ) groups.

Figure 3 illustrates the top 20 species by relative abundance at the phylum and genus levels. At the phylum level, the breast milk microbiota predominantly consists of Firmicutes, Proteobacteria, Actinobacteriota, and Bacteroidota. The percentage of these phyla was 85% in the HC group and 75.1% in the NJ group (Figure 3A). Although there were differences in the abundance of these phyla between the two groups, these differences were not statistically significant. At the genus level, *Staphylococcus* and *Streptococcus* were the most abundant genera in breast milk. Other genera with relative abundances greater than 1% in both groups included *Gemella*, *Acinetobacter*, *Vibrio*, *Pseudoalteromonas*, *Cutibacterium*, *Rothia* and *Ralstonia* (Figure 3B). Particularly, *Lactobacillus* was found to be more abundant in the HC group (HC 1.34%, NJ 0.73%) compared to the NJ group, with a significant difference observed (*P* < 0.01).

#### 3.2.3 Differential analysis of breast milk microbiota

**Figure 4:**
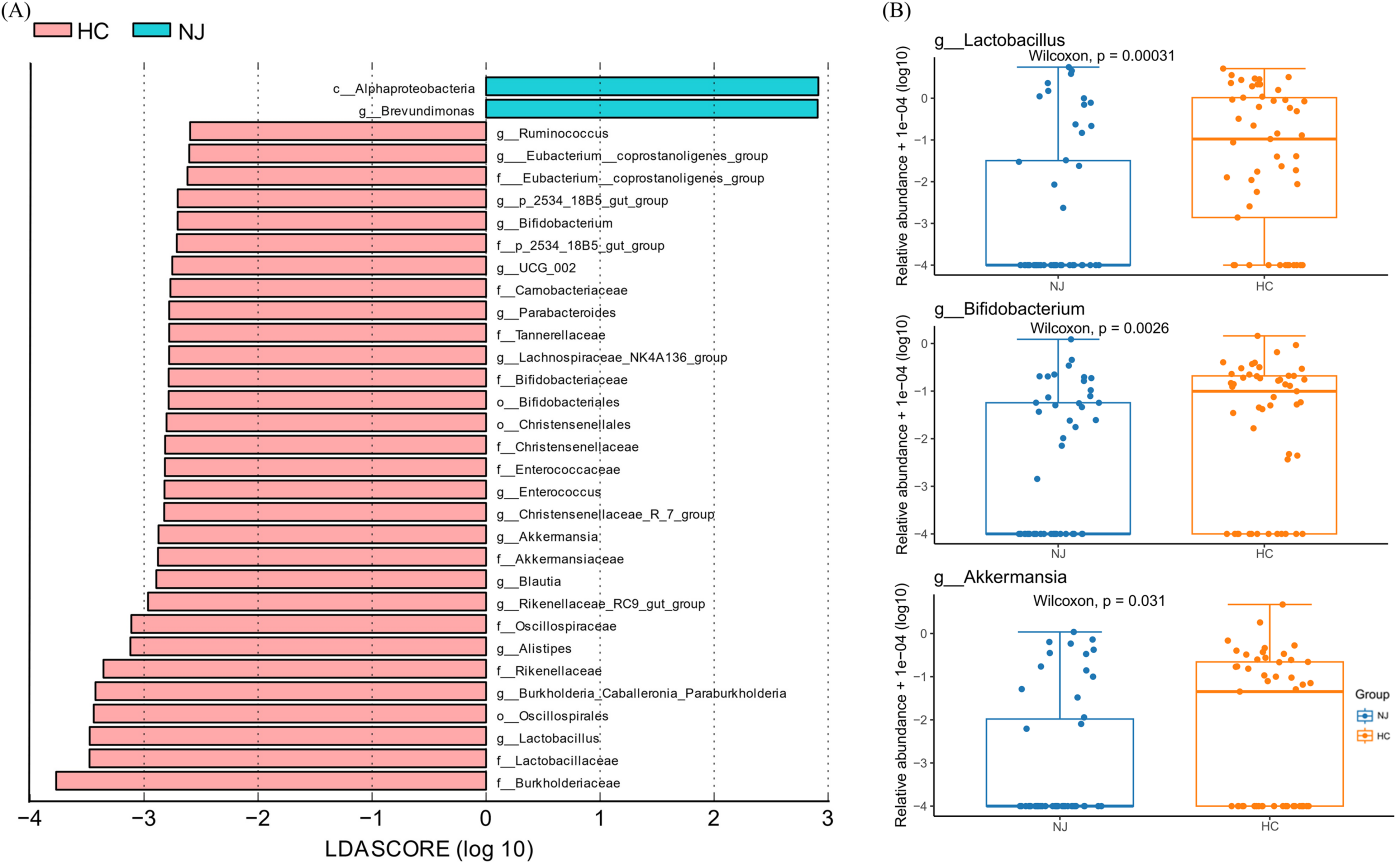
Differential analysis of breast milk microbiota between healthy control (HC) and neonatal jaundice (NJ) groups. **(A)** LEfSe analysis between the HC and NJ groups, showing species with absolute values of linear discriminant analysis (LDA) score >2.5. **(B)** Differences in relative abundance of *Lactobacillus*, *Bifidobacterium*, and *Akkermansia* between HC and NJ groups.

To further investigate the differences in species between the two breast milk microbiota groups, we employed the LEfSe method to identify characteristic microorganisms. Species with a linear discriminant analysis (LDA) threshold greater than 2.5 were considered potential biomarkers. Differential species between the HC and NJ groups were predominantly observed at the family and genus levels, with a focus on genera (Figure 4A). We observed that characteristic microorganisms were more abundant in the HC group compared to the NJ group. Notably, *Brevundimonas* was more prevalent in the NJ group. The HC group had higher levels of *Lactobacillus*, *Burkholderia_Caballeronia_Paraburkholderia*, *Alistipes*, *Rikenellaceae_RC9_gut_group*, *Blautia*, *Akkermansia*, *Christensenellaceae_R_7_group*, *Enterococcus*, *Lachnospiraceae_NK4A136_group*、*Parabacteroides*, *UCG-002*, *Bifidobacterium*, *p_2534_18B5_gut_group*, *Eubacterium__coprostanoligenes_group,* and *Ruminococcus* was higher in breast milk in the HC group (*P* < 0.05). Among them, *Lactobacillus*, *Bifidobacterium* and *Ackermannia* were common beneficial bacteria in breast milk, and these three beneficial bacteria were significantly higher in the breast milk of the cases from the HC group (Figure 4B).

### 3.3 Analysis of breast milk metabolites

**Figure 5:**
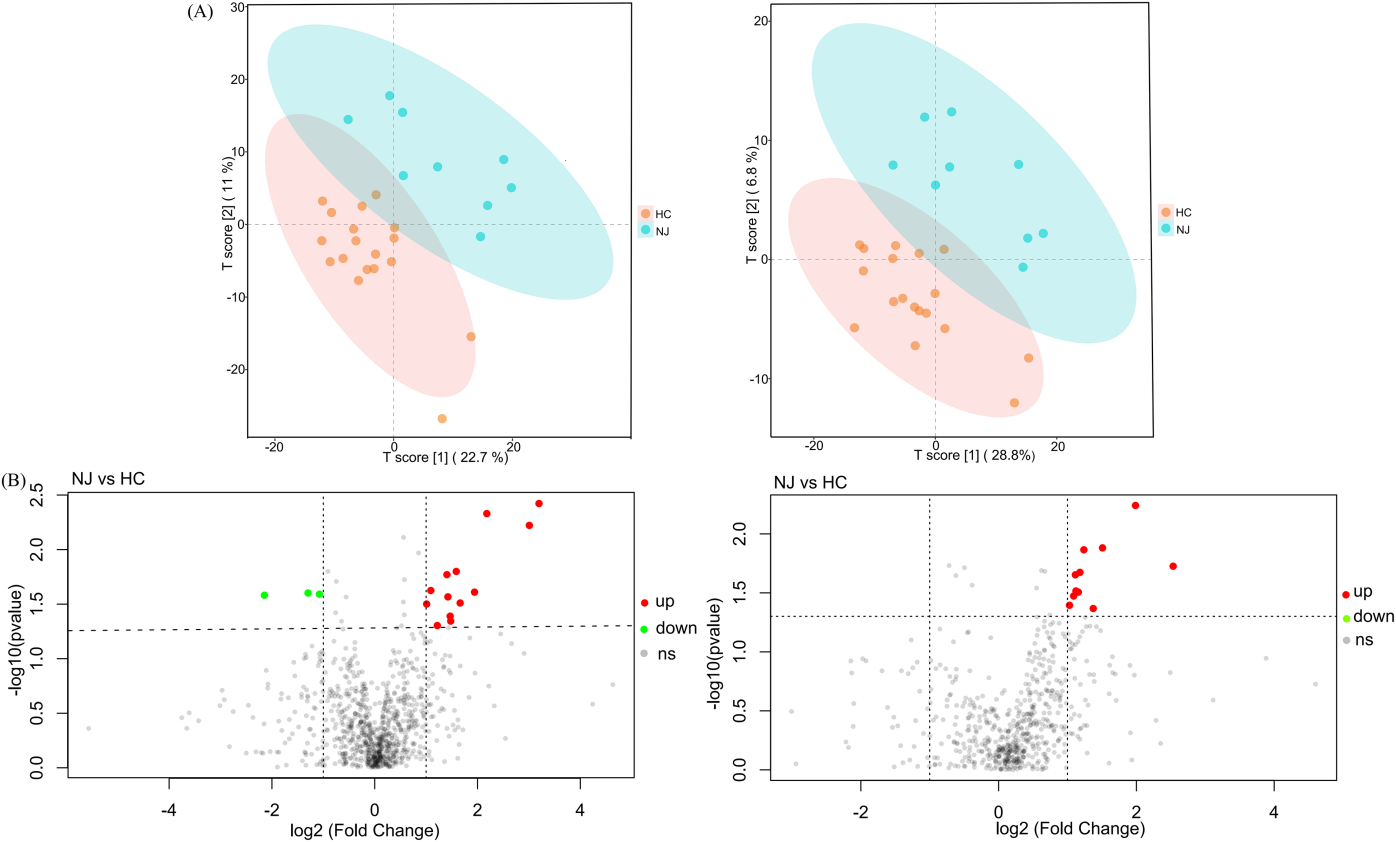
PLS-DA models and differential metabolite volcano maps for healthy control (HC) and neonatal jaundice (NJ) groups. **(A)** Score plot of the PLS-DA model in ESI+ and ESI- ion modes for HC and NJ groups. **(B)** Volcano map of differential metabolites in ESI+ and ESI- ion modes for HC and NJ groups. Each point represents a metabolite, horizontal coordinates representing the fold change (log2FoldChange), and vertical coordinates representing the significance level (-log10P value). Metabolites that were significantly up-regulated in the HC group compared to the NJ group are indicated in red, and those were significantly down-regulated are indicated in green.

We analyzed 18 breast milk samples from the HC group and 9 samples from the NJ group using a non-targeted metabolomics approach based on LC-MS. The PLS-DA model revealed significant clustering in both ESI+ and ESI- ion modes, indicating notable differences in metabolite profiles between the two groups (Figure 5A). In ESI+ ion mode, we identified a total of 867 metabolites, while in ESI- ion mode, we detected 600 metabolites. Overall, 27 differential metabolites were identified between the two groups: 16 in ESI+ ion mode and 11 in ESI- ion mode (Figure 5B). In particular, 24 metabolites, predominantly lipids, were significantly elevated in the HC group compared to the NJ group (Table 2). This includes members of the sphingolipid family [e.g., SPB 17:1;2O, SM 9:1;2O/24:6, D-Glucosyl-beta-1,1-N-palmitoyl-D-erythro-sphingosine, N,N-Dimethylsphingosine, Sphingosine (d18:1), D-Sphingosine], the phospholipid family (e.g., PC O-34:1, PC O-36:4, PC 19:1_19:2, PC O-22:5, PS 18:1_18:1, LPC 20:0-SN1, LPC O-15:0, LPC 22:5, LPC 20:3), and the fatty acid derivatives family (e.g., FAHFA 6:0/18:2, FAHFA 18:1/20:2, (±)10(11)-EpDPA, (±)8(9)-DiHET). Conversely, three metabolites—piperine, 3-Nitro-L-tyrosine, and cytidine—were significantly reduced in the HC group. These differential metabolites may play key roles in distinguishing between the two subgroups.

**Table 2:** Differential metabolites^a^ in healthy control (HC) and neonatal jaundice (NJ) groups.

Name	Fold Change^b^	Type	*P*-value
PC O-34:1	9.16	Up	0.004
PC O-36:4	8.04	Up	0.006
LPC 20:0-SN1	4.53	Up	0.005
Piperine	0.23	Down	0.026
PS 18:1_18:1	3.84	Up	0.025
PC 19:1_19:2	3.17	Up	0.031
SPB 17:1;2O	3.00	Up	0.016
L-beta-Imidazolelactic acid	2.79	Up	0.045
Urocanic acid	2.77	Up	0.041
N,N-Dimethylsphingosine	2.68	Up	0.027
PC O-22:5	2.65	Up	0.017
3-Nitro-L-Tyrosine	0.40	Down	0.025
Sphingosine (d18:1)	2.32	Up	0.049
D-Sphingosine	2.13	Up	0.024
Cytidine	0.48	Down	0.026
SM 9:1;2O/24:6	2.01	Up	0.032
D-Glucosyl-beta-1,1-N-palmitoyl-D-erythro-sphingosine	5.79	Up	0.019
LPC O-15:0	3.96	Up	0.006
LPC 22:5	2.85	Up	0.013
FAHFA 6:0/18:2	2.59	Up	0.043
(±)10 (11)-EpDPA	2.36	Up	0.014
N-Phenylacetylglutamine	2.27	Up	0.021
5′-O-Methylthymidine	2.23	Up	0.031
(±)8 (9)-DiHET	2.18	Up	0.030
LPC 20:3	2.17	Up	0.022
FAHFA 6:0/18:2	2.13	Up	0.034
D-Fucose	2.04	Up	0.040

^a^ Select the metabolites with fold change ≥2 and fold change ≤0.5. The difference of metabolites between group HC and group NJ was more than 2 times or less than 0.5, and the difference was considered to be significant. “up” represents up-regulation of metabolite content; “down” represents down-regulation of metabolite content.

^b^ Utilizing the NJ group as a reference, the fold change (FC) in the mean expression level (E) for the HC group, in comparison to the mean expression level (C) of the NJ group, was computed according to the equation $FC=\frac{E}{C}$. In volcano plots, the fold change values are represented in logarithmic form as log2 (FC). A positive log2 (FC) value signifies an elevation in the expression level of the HC group compared to the NJ group, whereas a negative value denotes a reduction in the expression level of the HC group relative to the NJ group.

## 4 Discussion

### 4.1 Association of breast milk microbiota with neonatal jaundice

We used 16S rRNA sequencing to identify microbiota in breast milk from healthy newborns and jaundiced mothers. Alpha diversity analysis and beta diversity analysis showed that there was no difference in the total abundance and diversity of breast milk microbiota between the two groups. At the genus level, we observed a relatively higher abundance of *Lactobacillus*, *Bifidobacterium*, and *Ackermannia* in the breast milk of the healthy control group compared to the neonatal jaundice group. This suggesting a potential association between these bacterial taxa and the development of neonatal jaundice. These beneficial bacteria in breast milk can be passed vertically to the neonate through breastfeeding and are essential for establishing and regulating the neonatal intestinal flora, which may provide some protection against pathogen invasion and reduce inflammatory responses (8, 36). Wang et al. (37) found that *Bifidobacterium*, *Ackermannia*, *Ruminococcus*, and other beneficial bacteria in breast milk may protect infants from food allergies by influencing intestinal flora homeostasis. Specific *Lactobacillus* species not only enhance mucosal defense barriers in neonates but also modulate their immune responses through various mechanisms, thus providing immune protection (38, 39). Furthermore, the combination of blue light therapy and *Lactobacillus rhamnosus* GG has been shown to accelerate the expulsion of fetal stool in neonatal jaundice, reducing the duration of phototherapy (40). Aromatic lactic acid, produced by *Bifidobacterium* species in the intestinal tract of newborns, can directly influence immune cells, thereby enhancing immune responses during early life (26, 41, 42). Jaundiced infants have been shown to have lower concentrations of *Bifidobacterium* in both breast milk and feces compared to healthy breastfed newborns, which aligns with our results (43). An increase in β-glucuronidase activity leads to enhanced enterohepatic circulation of bilirubin, which subsequently elevates bilirubin levels in the blood, a key factor in the development of neonatal jaundice (44, 45). *Bifidobacterium* has been reported to inhibit bilirubin enterohepatic circulation by promoting defecation, lowering intestinal pH, and decreasing β-glucuronidase activity, thus favouring the metabolism of bilirubin to a certain extent (19, 46). Moreover, the addition of *Bifidobacterium animalis* subsp. *lactis* CP-9, isolated from breast milk, during phototherapy has been found to synergistically enhance the therapeutic efficacy for neonatal jaundice by reducing the total duration of phototherapy and increasing the rate of serum bilirubin reduction (47). In recent years, *Ackermannia* has been shown to be a beneficial intestinal bacterium that is present in the breast milk microbiota and plays an important role in the establishment of the neonatal intestinal microbiota (48). This microorganism shows potential for modulating diseases, enhancing neonatal intestinal barrier function, and reducing inflammatory responses (49, 50). These effects of improving host health may indirectly contribute to the development of neonatal jaundice. Furthermore, Chen et al. (20) concluded from a systematic review and meta-analysis that enteral probiotic supplementation supports their colonization in the gastrointestinal tract and is both safe and effective for the treatment of neonatal jaundice. However, these studies were conducted in the context of combined phototherapy. There is insufficient evidence that probiotic strains of *Bifidobacterium* and *Lactobacillus* species have a direct beneficial effect on bilirubin metabolism (51). This suggests that future studies should design cellular or animal model experiments to assess the effects of probiotics alone on bilirubin metabolism, both *in vitro* and *in vivo*, and to investigate the mechanisms of action.

Beneficial bacteria can be passed on to the infant through breastfeeding and can colonise the infant's gut. In the neonatal intestinal tract, these beneficial bacteria may reduce the risk of neonatal jaundice by reducing hepatic and intestinal bilirubin circulation, through mechanisms such as balancing bacterial flora, enhancing immune function and reducing β-glucuronidase activity. Although our observations indicated that these beneficial microorganisms are generally present in low abundance in breast milk, certain critical metabolites produced by these low-abundance populations can reach high concentrations in the gut and bloodstream, significantly influencing physiological functions (52, 53). Thus, the role of beneficial microorganisms in breast milk, even at low levels, in the growth and development of breastfed infants should not be underestimated.

### 4.2 Association of breast milk metabolites with neonatal jaundice

Small molecule metabolites in breast milk are crucial for neonatal growth and development, yet research on their presence and functions remains limited (54, 55). Using LC-MS, we detected a broad spectrum of small molecule metabolites in breast milk, encompassing lipids, organic acids, carbohydrate, nucleic acids, and others. These substances collectively offer comprehensive nutritional support and health protection for newborns during their early developmental stages. In this present study, we compared metabolite levels in the breast milk of healthy and jaundiced infants. We observed significant differences in metabolite concentrations, with notably higher levels of lipids—specifically sphingolipids, phospholipids, and fatty acid derivatives—in the breast milk of healthy infants. Lipids are integral components of the milk fat globule membrane (MFGM) and exert a range of biological effects, making them key contributors to the beneficial properties of breast milk. Breast milk lipids play an important role in gastrointestinal function, infant growth, neurodevelopment and immunity (56–58). Phospholipids and sphingolipids are important polar lipids in milk and are mainly found in the MFGM. They play an indispensable role in neonatal nutrition, not only by promoting intestinal development and maturation, but also by significantly improving the composition of the intestinal microbiota (59). Currently, the study of milk polar lipids and their metabolites is focused on the MFGM form. Gong et al. (60) found that the addition of MFGM to formula accelerated the maturation of the intestinal mucosal barrier by promoting intestinal proliferation and differentiation and increasing the expression of tight junction proteins in rat pups. In addition, MFGM-supplemented formula-fed pups had an intestinal flora composition closer to that of breast-fed pups than formula-fed pups alone. Li et al. (61) showed that the structure of the gut microbial community in mice fed a high-fat diet could be modulated by MFGM supplementation, thereby ameliorating the gut inflammatory response associated with obesity.

The sphingolipid family, which includes sphingomyelin, glycosphingolipids, and sphingosine, plays a critical role in the immune development of infants. In a 4-week mouse study, mice consuming milk SM had an increased abundance of Bacteroidaceae and *Bifidobacterium* in their faeces, a change that may help mitigate the negative effects of a high-fat diet (62). At the same time, milk SM may also play a role in protecting the intestinal tract from inflammation, and the addition of 0.1% milk SM to the diet can alleviate intestinal inflammation in a mouse model of colitis (63). In addition, sphingomyelin and glycosphingolipids are digested and absorbed in the intestinal where they produce bioactive metabolites such as ceramide, sphingosine, and sphingosine-1-phosphate. These metabolites are essential for maintaining intestinal integrity and supporting the development of the neonatal immune system (64). Sphingosine is rapidly absorbed by the neonatal intestinal mucosa and is extensively converted into palmitic acid, an unsaturated fatty acid critical for the organism's healthy development (65). Phospholipids are essential for several biological functions, including maintaining the structural integrity of cell membranes, transmitting cellular signals, promoting cell proliferation, and modulating inflammatory responses (59). The benefits of phospholipids for gut health are reflected in their interactions with microorganisms. A study showed that phospholipids interacting with *Lactobacillus* can significantly affect *Lactobacillus* adhesion, thereby improving the health of the gut microbiota (66). Zhao et al. (67) showed that phospholipids were significantly positively correlated with the abundance of the beneficial bacterium *Bifidobacterium* and negatively correlated with the abundance of the conditionally pathogenic bacterium *Escherichia/Shigella* in the infant gut. Additionally, Wang et al. (68) demonstrated that phospholipids effectively reduced sodium dextran sulfate-induced intestinal inflammation in mice through the administration of phospholipid concentrates. There is an association between breast milk fatty acids and the development of neonatal jaundice. Studies have shown that high concentrations of fatty acids (e.g., oleic acid, linoleic acid, DHA) promote the expression of UDP-glucuronosyltransferase 1A1 in the liver and small intestine, leading to a decrease in serum bilirubin levels in newborns (69). However, the relationship between fatty acid derivatives and neonatal jaundice is currently unproven. However, the fatty acid hydroxy fatty acid (FAHFA) family, present in breast milk (70), has been shown to reduce the production of inflammatory cytokines and exhibit strong anti-inflammatory properties (71). Breast milk lipids are transferred to the neonatal gut through breastfeeding. These lipid components may influence the development of neonatal jaundice by regulating the neonatal intestinal flora and exerting anti-inflammatory effects to maintain a healthy intestinal environment. Our study represents the first investigation into the association between metabolites in breast milk and neonatal jaundice. While there is no direct evidence that the lipid family in breast milk specifically protects against neonatal jaundice, the beneficial roles of these substances in neonatal growth and development suggest they may indirectly influence the condition. Further research is needed to verify these findings and elucidate the specific mechanisms by which these metabolites may affect neonatal jaundice.

### 4.3 Study limitations

Breastmilk is essential for the healthy development of newborns and serves as a primary reference for formula design. However, studying the composition of breast milk and its role in neonatal growth and development presents significant challenges. This study is the first to explore the association between jaundice in breastfed newborns and the microbiota and small molecule metabolites in breast milk. Despite such a promising approach, several limitations must be acknowledged. First, while we aimed to homogenize the composition of breast milk when analyzing its flora and metabolites, the inherent complexity of breast milk and potential interactions among its various components may affect our results. Second, our study exclusively analyzed breast milk samples from mothers and did not include fecal samples from the newborns. Furthermore, as there are significant compositional differences between foremilk, hindmilk and whole milk, and we only analysed foremilk samples, this may not adequately represent the full composition of the flora and metabolites in breast milk. Consequently, we could not fully assess how breast milk influences the intestinal flora of newborns and its impact on the development of jaundice. Additionally, the sample size in our study was relatively small. A larger sample size is typically required to accurately determine the composition of breast milk and the role of its specific components, ensuring the generalizability and precision of the findings. Future research should address these limitations by incorporating a larger sample size and employing multi-omics techniques to more comprehensively investigate the relationship between breastfeeding and neonatal jaundice, as well as the role of various breast milk components in the development of jaundice.

## 5 Conclusions

This study examined the association between neonatal jaundice and the microbiota and small molecule metabolites in breast milk from exclusively breastfed mothers and their infants. Our findings indicate that breast milk from mothers of healthy newborns contains higher levels of beneficial bacteria (*Lactobacillus*, *Ackermannia*, and *Bifidobacterium*) and lipid families (sphingolipids, phospholipids, and fatty acid derivatives) compared to that from mothers of jaundiced infants. These substances are passed on to newborn through breastfeeding and can affect the development of neonatal jaundice. They can reduce the enterohepatic circulation of bilirubin by promoting the establishment of a healthy neonatal intestinal microbiota, reducing inflammatory response and boosting immunity, which in turn reduces the risk of neonatal jaundice. This study offers new insights into the relationship between breastfeeding and neonatal jaundice, highlighting the potential benefits of specific breast milk components.

## Data Availability

Publicly available datasets were analyzed in this study. This data can be found here: The data supporting the findings in this manuscript are available from the corresponding authors upon reasonable.
